# Changes in Choroidal Structures in Eyes with Chronic Central Serous Chorioretinopathy after Half-Dose Photodynamic Therapy

**DOI:** 10.1371/journal.pone.0163104

**Published:** 2016-09-16

**Authors:** Takamasa Kinoshita, Yoshinori Mitamura, Terumi Mori, Kei Akaiwa, Kentaro Semba, Mariko Egawa, Junya Mori, Shozo Sonoda, Taiji Sakamoto

**Affiliations:** 1 Department of Ophthalmology, Institute of Biomedical Sciences, Tokushima University Graduate School, Tokushima, Japan; 2 Department of Ophthalmology, Sapporo City General Hospital, Sapporo, Japan; 3 Department of Ophthalmology, Kagoshima University Graduate School of Medical and Dental Sciences, Kagoshima, Japan; Massachusetts Eye & Ear Infirmary, Harvard Medical School, UNITED STATES

## Abstract

**Purpose:**

To determine the structural changes in the choroid after half-dose photodynamic therapy (hPDT) in eyes with chronic central serous chorioretinopathy (CSC).

**Methods:**

This was a retrospective interventional study of 29 eyes of 29 patients who underwent hPDT for chronic CSC with serous retinal detachment (SRD) and were followed for ≥3 months. Enhanced depth imaging optical coherence tomographic (EDI-OCT) images of the subfoveal choroid were converted to binary images. The central choroidal thickness (CCT), the cross sectional subfoveal choroidal area, the hyporeflective and hyperreflective areas of the inner, outer, and whole choroid were determined at the baseline, and at 1, 3, and 12 months after the hPDT.

**Results:**

The SRDs were resolved in 26 (89.7%) eyes at 3 months after the hPDT. The mean CCT (*P* = 0.001), the total choroidal area (*P* = 0.001), and the hypo-reflective area (*P* = 0.003) of the whole choroid were significantly decreased from the baseline at 3 months. The hyperreflective area of whole choroid was not significantly changed during the study period (*P* = 0.083). The hyperreflective but not the hyporeflective area of the inner choroid was significantly decreased at 3 months (*P* = 0.001, *P* = 1.000, respectively). The hyporeflective but not the hyperreflective area of the outer choroid was significantly decreased at 3 months (*P* = 0.001, *P* = 1.000, respectively).

**Conclusions:**

The hyperreflective area of the inner choroid and hyporeflective area of the outer choroid were significantly decreased after hPDT for chronic CSC. Because the hyperreflective and hyporeflective area correspond to the choroidal stroma and vessels, respectively, the decreased CCT and subfoveal choroidal area after hPDT may be attributed to a decrease in the exudative changes in the inner choroidal stroma and the reduction of the dilation of the outer choroidal vessels.

## Introduction

Recent studies using enhanced depth imaging optical coherence tomography (EDI-OCT) or swept-source OCT have shown that the choroid is closely associated with the pathogenesis of various retinal diseases [1–6]. Central serous chorioretinopathy (CSC) is believed to be one of those diseases in which abnormalities in the choroid such as an abnormal ocular circulation and an increased vascular permeability can cause a thickening of the choroid and an increase in the hydrostatic pressure which can lead to a disruption of the retinal pigment epithelium and the development of a serous retinal detachment (SRD) [1,2,7–11]. Although CSC usually resolves spontaneously in a few months, persistent or recurrent SRDs can lead to a permanent reduction of vision [12,13].

Photodynamic therapy (PDT) has been shown to be effective in treating chronic CSC [14–16], and modified PDT such as half-dose PDT (hPDT) [17–21] or reduced-fluence PDT [19,22–25] led to favorable results with less complications. The thickening of the choroid decreases after hPDT [2,25]. However, because a layer-by-layer architecture is not distinct in choroid, the changes in detailed choroidal structures except for the thickness and area have not been determined in these eyes.

We have recently reported on a new method to differentiate and quantify the choroidal lumens from the stroma using an open access software named ImageJ [26–33]. Earlier studies and many empirical observations strongly suggested that the hyporeflective areas in the EDI-OCT images represented the luminal or fluid-filled areas and that the hyperreflective areas represented the stromal areas [34–36]. We used the binarization technique to differentiate the choroidal luminal area from the stromal area, and we reported that not only the choroidal thickness but also the ratio of luminal area to total choroidal area decreased significantly with aging and longer axial lengths in normal eyes [27]. The choroidal thickness and the ratio of the luminal area to the total choroidal area also decreased after PDT in eyes with age-related macular degeneration and after corticosteroidal therapy in eyes with Vogt-Koyanagi-Harada disease [26,28].

In eyes with CSC, the ratio of the hyporeflective area to the total choroidal area was significantly larger than that of normal eyes, and the ratio in the fellow eyes of the patients with unilateral CSC was significantly larger than that of normal eyes [33,37]. Knowledge of the sizes and ratios of the luminal and stromal areas in CSC eyes before and after hPDT should be useful for understanding the pathogenesis of CSC.

Thus, the purpose of this study was to determine the changes in the hyporeflective and hyperreflective areas of the choroid in eyes with chronic CSC before and after hPDT by binarizing of the EDI-OCT images.

## Methods

The procedures used conformed to the tenets of the Declaration of Helsinki, and a written informed consent was obtained from all of the patients after the intent of the study had been fully explained. An approval was obtained from the Institutional Review Board of Tokushima University Hospital for the study protocol.

### Inclusion and exclusion criteria

This was a retrospective, consecutive case series of 29 eyes of 29 patients who underwent hPDT for a chronic CSC at the Tokushima University Hospital between April 2013 and April 2016. The eyes with chronic CSC were defined as eyes with a SRD that was detected by spectral-domain OCT (SD-OCT) to have persisted for more than 6 months with areas of alterations in the retinal pigment epithelium such as depigmentation or retinal pigment epithelial detachment. Fluorescein angiography (FA) of these eyes showed diffuse or multiple areas of granular hyperfluorescence and indistinct leakage [38].

The inclusion criteria were patients with chronic CSC associated with a SRD involving the fovea who had undergone hPDT and were followed for at least 3 months. Forty-one eyes of 39 patients were screened for the possible enrollment for the study. Twelve eyes were excluded from the study. The exclusion criteria included; (1) best-corrected visual acuity (BCVA) >1.0 logarithm of the minimum angle of resolution (logMAR) units (1 eye), (2) eyes with polypoidal vasculopathy (3 eyes), (3) high myopia defined as a refractive error (spherical equivalent) < -6.0 diopters or an axial length >26.5 mm (1 eye), (4) patients under corticosteroid therapy (1 eye), (5) patients with other ocular diseases that may affect the visual acuity or clinical findings (1 eye), (6) patients who had undergone intraocular surgeries including laser photocoagulation (3 eyes), and (7) patients who received hPDT for bilateral chronic CSC (4 eyes). Two of 4 eyes treated with hPDT for bilateral chronic CSC had also undergone laser photocoagulation. There were 2 patients with diabetes mellitus. These two patients were excluded because of a moderate cataract and a history of intraocular surgery. There were 2 smokers in the 39 patients screened. These two patients were excluded because of polypoidal vasculopathy and bilateral chronic CSC treated with hPDT. Twelve patients with well-controlled hypertension and 4 patients with well-controlled hyperlipidemia were included. In the end, 29 eyes of 29 patients were studied. The untreated fellow eyes without SRD were used as controls.

### Ophthalmic examinations

The ophthalmic examinations used for the diagnosis of CSC included measurements of the BCVA and the intraocular pressure, slit-lamp biomicroscopy, indirect ophthalmoscopy, fundus photography, FA (TRC50DX, TOPCON, Tokyo, Japan), indocyanine angiography (IA; TRC50DX, TOPCON), and SD-OCT (Spectralis OCT, Heidelberg Engineering, Heidelberg, Germany). The measurements were performed before and at 1, 3, and 12 months after the half-dose PDT. FA and IA were performed at the baseline. IA was performed to identify the areas of choroidal hyperpermeability which was used for the diagnosis of chronic CSC, and also as a guide of the size and location to apply the hPDT. IA was also used to detect polypoidal lesions, and cases of polypoidal choroidal vasculopathy were excluded. The degree of hyperfluorescence in the middle phase of IA was classified as intense or intermediate hyperfluorescence according to the classification by Inoue et al [24]. The BCVA was measured with a standard Japanese Landolt visual acuity chart, and the decimal visual acuity was converted to logMAR units for statistical analyses. All baseline data were obtained within two weeks of the hPDT.

### Spectral domain optical coherence tomography (SD-OCT)

SD-OCT was performed at each visit with the Heidelberg Spectralis OCT instrument at each visit. Cross sectional images of 30 degrees through the fovea were obtained by EDI-OCT for each eye. Each image was obtained through a dilated pupil using the eye tracking system, and 100 scans were averaged to improve the signal-to-noise ratio. Three horizontal and 3 vertical scans through the central fovea were performed at each visit by an experienced orthoptist who was masked to the patients’ clinical findings, and the best images in each direction were selected by one of the authors (ME) in a masked fashion to be used for the analyses. All images were obtained between 11:00 and 13:00 hours to minimize the effect of the diurnal variations in the choroidal structures [32].

The parameters measured were the height of the SRD, the thickness of the neurosensory retina at the central fovea, the subfoveal choroidal thickness, total choroidal area, and the hyporeflective and hyperreflective areas of the choroid. The ratio of the hyporeflective area to the total choroidal area was calculated. The examined area of the subfoveal choroid was 1500 μm wide. The height of the SRD was set to be the distance between the outer border of the detached neurosensory retina and the inner surface of the RPE. The thickness of the neurosensory retina was defined as the distance between the vitreoretinal interface and the outer border of the detached neurosensory retina, and the central choroidal thickness (CCT) as the distance between the outer border of the RPE and the chorioscleral interface. These distances were measured by two independent investigators (ME and KS) using the built-in measuring software in the Heidelberg Spectralis OCT instrument. The averages of two measurements were used for the statistical analyses.

### Evaluation of total choroidal area, hyporeflective and hyperreflective area by binarization technique

The selected best images were evaluated by one of our authors (ME) in a masked fashion. The binarization of the choroidal area in the EDI-OCT images was performed by a modified Niblack method as described in detail [26,30]. Briefly, an EDI-OCT image was analyzed by freely available software (ImageJ version 1.47, NIH, Bethesda, MD, USA). The subfoveal choroidal area of 1,500 μm wide that extended vertically from the retinal pigment epithelium to the chorioscleral border was examined. This area was selected with the ImageJ ROI Manager. Three choroidal vessels with lumens >100 μm were randomly selected by the Oval Selection Tool on the ImageJ tool bar, and the reflectivities of these lumens were averaged. The average reflectivity was set as the minimum value to reduce the noise in the OCT image. Then, the image was converted to 8 bits and adjusted by the Niblack Auto Local Threshold. The binarized image was converted to the RGB image again. These conversions were technical requirements for the binarization procedures and the automated calculation by Image J. The hyporeflective area was determined using the Threshold Tool. The dark pixels were defined as the hyporeflective areas, and the light pixels were defined as the hyperreflective areas. After adding the data on the relationship between the distance on the fundus and the pitch of the pixels in the EDI-OCT images, which depends on the axial length, the hyperreflective and hyporeflective areas were automatically calculated.

**Fig 1:**
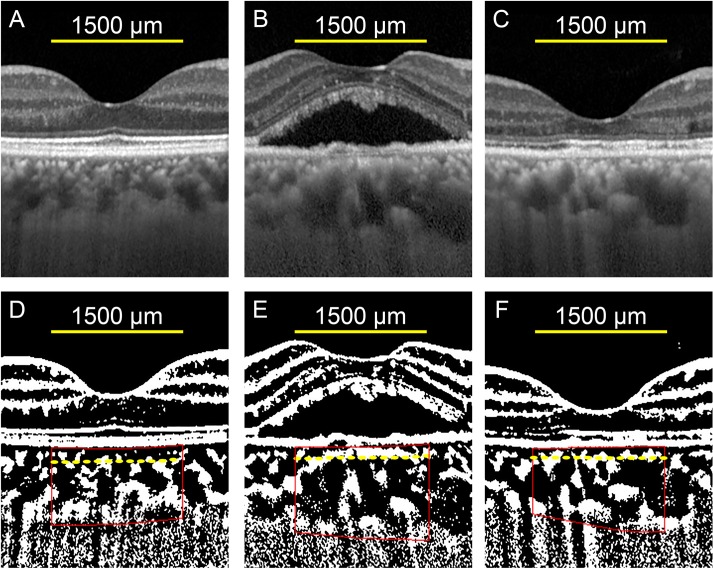
Representative enhanced depth imaging optical coherence tomographic (EDI-OCT) images and the converted binary images of the eye of a 55-year-old man with central serous chorioretinopathy (CSC). (A, B, C) EDI-OCT images of the fellow eye (A), treated eye at baseline (B), and at 3 months after hPDT (C). (D, E, F) The converted binary images of the EDI-OCT images shown in A (D), B (E), and C (F). The margins of the region of interest (ROI) are delineated by red lines. Yellow dashed lines indicate the border of the inner and the outer choroid.

Evaluations of the choroidal areas were also performed for the inner choroid and the outer choroid. The inner choroid included the choriocapillaris and medium choroidal vessel layer, and the outer choroid included the larger choroidal vessel layer. The margins of the inner and the outer choroid were determined by the Branchini et al method with some modifications [35]. The segmentation was done on the binarized OCT images to enhance the margin of the larger choroidal vessels (Fig 1).

All parameters from the horizontal and vertical images were measured three times, and the averages of the 6 measurements were used for the data analyses. Although the methods used for the analysis of the choroid was found to have high repeatability and reproducibility in normal eyes and eyes with CSC [26, 33, 37], the intra-rater correlation coefficient was calculated for the choroidal areas in the EDI-OCT images of CSC eyes at the baseline.

### Half-dose photodynamic therapy

Half-dose PDT (hPDT) was performed when a SRD involving fovea was present or was estimated to be present for more than 6 months. Whether a decrease in the visual acuity had occurred was not considered as an indication of hPDT. It was done by administering 3 mg/m^2^ of verteporfin (Visudyne; Novartis, Switzerland) intravenously over a period of 10 minutes as compared with the normal dose of 6 mg/m^2^ [18]. Fifteen minutes after the beginning of the verteporfin infusion, the selected area of the eye was exposed to a 689 nm laser light with an intensity of 600 mW/cm^2^ over 83 seconds for a total energy of 50 J/cm^2^. The treatment spot size was determined by the area of hyperpermeability in the IA images with an additional 1000 μm around the margin.

We measured the BCVA, height of the SRD, thickness of the neurosensory retina at the fovea, CCT, total choroidal area, and the hyporeflective and hyperreflective areas of the choroid before and 1, 3, and 12 months after the hPDT. The ratio of the hyporeflective area to the total choroidal area was calculated at each visit.

We have proposed an index called the CSC index that reflects the activity of the disease in eyes with CSC [33]. The formula of the CSC index was:

$$CSC\;index=\frac{\frac{hyporeflective\;area\;of\;outer\;choroid}{hyperreflective\;area\;of\;outer\;choroid}}{\frac{hyporeflective\;area\;of\;inner\;choroid}{hyperreflective\;area\;of\;inner\;choroid}}$$

We also evaluated the changes in the CSC index before and after the hPDT.

### Statistical analyses

Statistical analyses were carried out with the SPSS version 22 software (IBM, Armonk, New York, USA). The significance of the differences in the BCVA and the EDI-OCT parameters at the baseline and at 1 and 3 months was determined by repeated-measures analysis of variance with Greenhouse-Geisser corrections. The Bonferroni test was used for post hoc analyses. The significance of the differences between two paired samples was determined by paired *t* tests. The significance of the differences between two independent groups was determined by unpaired *t* tests. The intra-rater correlation coefficients were calculated by 1-way random effects model for measurements of agreement. A two-sided *P* value of <0.05 was considered statistically significant.

## Results

### Baseline demographic data

The baseline demographic data are shown in Table 1. One eye had received an intravitreous injection of anti-vascular endothelial growth factor agents more than 6 months before the hPDT. All patients underwent only 1 session of hPDT, and no other treatment including medical treatments was performed during the study period. All of the 29 eyes were followed for 3 months, and 22 eyes were followed for more than 12 months.

**Table 1:** Baseline Demographic Data of the Patients with Chronic CSC.

	Mean ± SD	Range
Sex	22 men / 7 women	
Age (yrs)	57.9 ± 9.26	44–82
Refractive Error (diopter)	-0.70 ± 2.00	-6.0 - +2.5
Duration of Symptom (median, months)	24	6–120
BCVA (logMAR), (number of letters)	0.16 ± 0.22, (75.72 ± 10.89)	-0.079–1.0 (35–85)
Hyperpermeability in IA	Intense 14 / intermediate 14	
Height of SRD	152.1 ± 82.35	22.5–339.5
Thickness of Neurosensory Retina	131.9 ± 39.09	73.5–246.0
Diameters of hPDT Treatment Size (μm)	4776.3 ± 1201.1	2150–6500

Values are the means ± SDs unless otherwise indicated.

CSC, central serous chorioretinopathy; SD, standard deviation; BCVA, best-corrected visual acuity; logMAR, logarithm of the minimum angle of resolution; IA, indocyanine angiography; SRD, serous retinal detachment; hPDT, half-dose photodynamic therapy

### Repeatability of measurement of OCT parameters

The intra-rater agreement was high with an intraclass correlation coefficient >0.9 for the measurements of all of the choroidal parameters (Table 2).

**Table 2:** Intra-rater agreement of measurements of choroidal area in eyes with CSC.

		ICC	95% CI	P-value
Whole Choroid	Total Area	0.997	0.995–0.999	P <0.001
	Hyporeflective Area	0.994	0.990–0.997	P <0.001
	Hyperreflective Area	0.934	0.878–0.967	P <0.001
Inner Choroid	Total Area	0.972	0.942–0.987	P <0.001
	Hyporeflective Area	0.988	0.975–0.994	P <0.001
	Hyperreflective Area	0.967	0.932–0.984	P <0.001
Outer Choroid	Total Area	0.994	0.988–0.997	P <0.001
	Hyporeflective Area	0.993	0.985–0.997	P <0.001
	Hyperreflective Area	0.914	0.821–0.959	P <0.001

CSC, central serous chorioretinopathy; ICC, intraclass correlation coefficients; CI, confidence interval

### Total, hyporeflective and hyperreflective choroidal areas at baseline

At the baseline, the mean CCT (*P* <0.001), the mean total choroidal area (*P* <0.001), the mean hyporeflective area (P <0.001), and the mean hyperreflective area (*P* = 0.025) of the whole choroid in the CSC eyes were significantly larger than those of the fellow eyes (Table 3). The mean ratio of the hyporeflective area to the total choroidal area in the treated eyes was significantly larger than that in the fellow eyes (*P* = 0.020). The mean hyperreflective area of the inner choroid of the CSC eyes was significantly larger than that in the fellow eyes (*P* <0.001). However, the hyporeflective area of the inner choroid was not significantly different from that of the fellow eyes (*P* = 0.153). The mean hyporeflective area of the outer choroid was significantly larger than that in the fellow eyes (*P* <0.001), but the mean hyperreflective area of the outer choroid was not significantly different from that of the fellow eyes (*P* = 0.578). A representative case is shown in Fig 1.

**Table 3:** Layer-by-layer analyses of choroidal structures of treatment eyes and the fellow eyes before and after half-dose PDT.

		Fellow eyes	Treated eyes					
		baseline	baseline		1M		3M	
		(N = 24)	(N = 29)	P-value^1^	(N = 22)	P-value^2^	(N = 29)	P-value^3^
CCT		310.9 ± 93.7	408.5± 107.9	**<0.001**	362.1 ± 105.9	**<0.001**	354.9 ± 110.6	**0.001**
Whole Choroid	Total Area	4.72 ± 1.34	6.14 ± 1.58	**<0.001**	5.45 ± 1.49	**<0.001**	5.36 ± 1.63	**0.001**
	Hyporeflective Area	3.24 ± 1.17	4.41 ± 1.40	**<0.001**	3.86 ± 1.24	**<0.001**	3.71 ± 1.41	**0.003**
	Hyperreflective Area	1.49 ± 0.23	1.73 ± 0.36	**0.025**	1.60 ± 0.32	0.075	1.65 ± 0.30	0.897
	Hyporeflective / Total Area (%)	67.1 ± 5.87	70.8 ± 5.51	**0.020**	69.9 ± 4.54	**0.030**	67.9 ± 5.65	**0.009**
Inner Choroid	Total Area	1.24 ± 0.30	1.33 ± 0.38	0.119	1.21 ± 0.32	0.106	1.21 ± 0.33	0.489
	Hyporeflective Area	0.83 ± 0.25	0.78 ± 0.27	0.153	0.76 ± 0.28	1.000	0.73 ± 0.27	1.000
	Hyperreflective Area	0.43 ± 0.11	0.55 ± 0.08	**<0.001**	0.45 ± 0.09	**0.003**	0.48 ± 0.10	**0.002**
	Hyporeflective / Total Area (%)	65.8 ± 7.22	57.1 ± 6.57	**<0.001**	61.1± 8.90	0.066	59.3 ± 7.95	**0.011**
Outer Choroid	Total Area	3.48 ± 1.11	4.78 ± 1.40	**<0.001**	4.25 ± 1.29	**0.001**	4.13 ± 1.39	**0.002**
	Hyporeflective Area	2.41 ± 0.98	3.61 ± 1.24	**<0.001**	3.11 ± 1.05	**0.001**	2.96 ± 1.21	**0.001**
	Hyperreflective Area	1.07 ± 0.21	1.12 ± 0.33	0.578	1.14 ± 0.31	1.000	1.17 ± 0.27	1.000
	Hyporeflective / Total Area (%)	67.3 ± 7.60	74.1 ± 6.29	**<0.001**	72.5 ± 4.49	**0.005**	70.2 ± 5.87	**0.003**

Values are the mean ± SDs (x 10^5^ μm^2^).

CSC, central serous chorioretinopathy; SD, standard deviation; CCT, central choroidal thickness

^1^Significance between fellow eyes and treated eyes at baseline

^2^Significance in the treated eyes between baseline and 1 month after the half-dose PDT

^3^Significance in the treated eyes between baseline and 3 months after the half-dose PDT

### Time course of changes in the BCVA and the retinal morphological parameters

**Fig 2:**
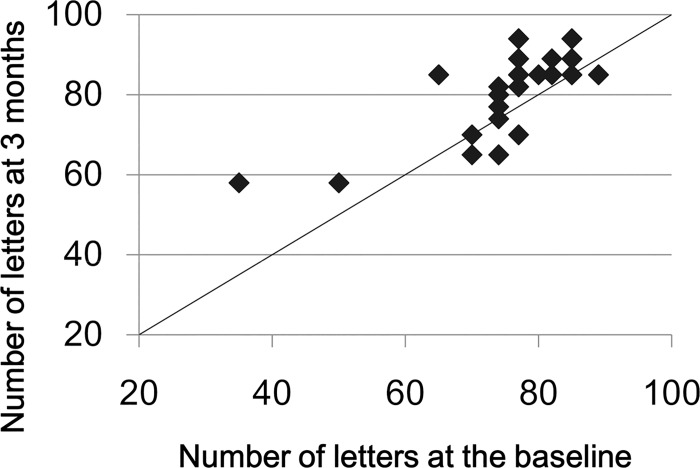
Scattered plots showing the associations between BCVA at the baseline and that at 3 months. No eye was worsened ≥10 letters at 3 months compared with that at baseline. The data of 7 eyes were overlapped each other in this scattered plots.

The mean BCVA at 3 months was 0.065 ± 0.204 logMAR units (81.7 ± 10.42 letters in ETDRS visual acuity charts) which was significantly better than that at the baseline (*P* = 0.022, Fig 2), and this improvement was maintained at 0.04 ± 0.172 (82.9 ± 8.76 letters) at 12 months. The SRD was completely resolved in 26 eyes (89.7%) at 3 months. Of the 3 eyes with SRDs at 3 months, the SRDs were still present in 2 eyes at 12 months, and 1 eye was lost to follow-up. No recurrence of the SRD was observed in the eyes for at least 12 months. The mean thickness of the neurosensory retina at 3 months was 147.4± 37.48 μm, which was significantly thicker than that at baseline (*P* = 0.038).

### Time course of changes in choroidal morphological parameters of whole choroid in treated eyes

**Fig 3:**
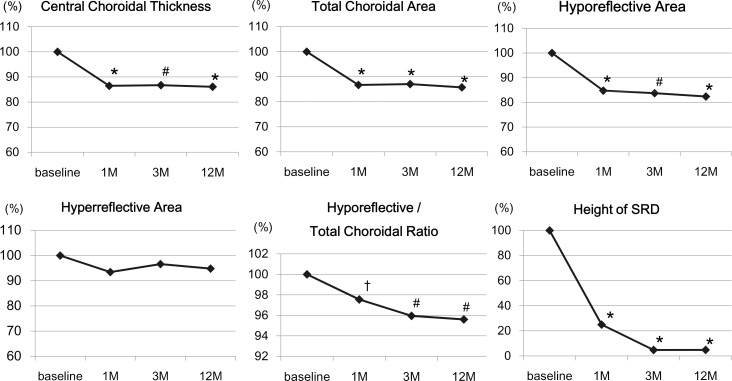
Time course of changes in EDI-OCT parameters. All parameters except the hyperreflective areas were significantly decreased from the baseline at 1 month and thereafter. Data are presented relative to that at the baseline. *P <0.001, #*P* <0.01, †*P* <0.05.

The mean CCT was significantly decreased at 1 month (*P* <0.001) and at 3 months (*P* = 0.001; Fig 3, Table 3, S1 Table). The mean CCT at 12 months was not significantly different from that at 3 months (*P* = 0.291). The mean total choroidal area of the whole choroid was significantly decreased from that at baseline at 1 month (*P* <0.001) and at 3 months (*P* = 0.001). The mean total choroidal area at 12 months was not significantly different from that at 3 months (*P* = 0.149).

The hyporeflective area of whole choroid was significantly decreased from that at the baseline at 1 month (*P* <0.001) and at 3 months (*P* = 0.003). However, the hyporeflective area of whole choroid at 12 months was not significantly different from that at 3 months (*P* = 0.090). There was no significant change in the mean hyperreflective area of the whole choroid during the study period (*P* = 0.083).

The ratio of hyporeflective area to choroidal area of whole choroid was significantly decreased from that at baseline at 1 month (*P* = 0.030) and at 3 months (*P* = 0.009). The ratio at 12 months was 67.5 ± 6.00% which was not significantly different from that at 3 months (*P* = 0.815). The ratios of normal controls was 65.2 ± 3.28% which was significantly lower than that of CSC eyes at the baseline (*P* <0.001, S2 Table), at 1 month (*P* <0.001), and at 3 months (*P* = 0.033), but was not significantly different from that at 12 months (*P* = 0.116).

### Time course of changes in different layers of choroid

**Fig 4:**
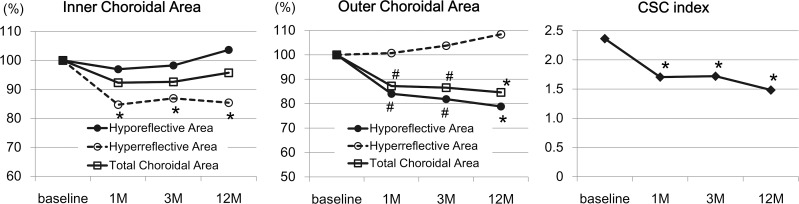
Time course of changes in EDI-OCT parameters in different layers of choroid In the inner choroid, the mean hyperreflective area but not the mean hyporeflective area was significantly decreased at 1 month and thereafter. In the outer choroid, the hyporeflective but not the hyperreflective area was significantly decreased at 1 month and thereafter. The CSC index was significantly decreased at 1 month and thereafter. The values of the choroidal areas are presented relative to that at the baseline. *P <0.001, #*P* <0.01.

In the inner choroid, there was no significant change in the total choroidal area and hyporeflective area during the follow-up periods (*P* = 0.103 and *P* = 0.718, Fig 4). However, the hyperreflective area of the inner choroid was significantly decreased from baseline at 1 month (*P* = 0.003) and at 3 months (*P* = 0.002). The ratio of the hyporeflective area to total choroidal area in the inner choroid was significantly increased from that at the baseline at 3 months (*P* = 0.011).

In the outer choroid, the total choroidal area was significantly decreased from that at the baseline at 1 month and at 3 months (*P* = 0.001 and *P* = 0.002). The hyporeflective area was also significantly decreased from the baseline value at 1 month and at 3 months (*P* = 0.001, both). However, there was no significant difference in the hyperreflective areas at any time during the 3-month follow-up period (*P* = 0.700). The ratio of the hyporeflective area to total choroidal area in the outer choroid was significantly decreased at 1 month (*P* = 0.005) and at 3 months (*P* = 0.003).

### Time course of changes in choroidal morphological parameters in the fellow eyes

There was no significant difference between all of the choroidal parameters of the whole, inner, and outer choroid at the baseline and those at 3 months in the untreated fellow eyes (*P* >0.100).

### The CSC index

At the baseline, the mean CSC index in the treated eyes was 2.36 ± 0.89 which was significantly higher than that in the 24 fellow eyes without a SRD at 1.24 ± 0.63 (*P* <0.001). The index was significantly reduced to 1.71 ± 0.60 at 1 month (*P* = 0.010), and 1.72 ± 0.60 at 3 months (*P* = 0.002). The index was 1.48 ± 0.60 at 12 months, which was significantly lower than that at 3 months (*P* = 0.035). The index at 12 months was not different from that in fellow eyes at baseline (*P* = 0.215), however, it was significantly larger than that in age-, sex-, and refractive error-matched normal controls (the index = 0.99 ± 0.37, *P* = 0.002, S2 Table).

### Relationship between choroidal morphological parameters and indocyanine green angiographic findings

At the baseline, the CCT, the total choroidal area, and the hyporeflective area of whole choroid were significantly larger in the eyes with intense hyperfluorescence in IA images than in the eyes with intermediate hyperfluorescence (*P* = 0.038, *P* = 0.046, and *P* = 0.033), The total choroidal area and the hyporeflective area of the outer choroid were significantly larger in the eyes with intense hyperfluorescence than in the eyes with intermediate hyperfluorescence (*P* = 0.015 and *P* = 0.021).

In the 3 eyes with a persistent SRD at 3 months, 2 eyes had intermediate hyperpermeability in the IA at the baseline, and one eye had intense hyperpermeability at the baseline. However, none of these distributions was significant (*P* = 1.000, Fisher’s exact test). There was no significant correlation between the presence of persistent SRD and the choroidal parameters.

## Discussion

At the baseline, the CCT and total choroidal area of the whole choroid were significantly larger in the treated eyes than in the fellow eyes. These changes were associated with increases in both the hyperreflective area of the inner choroid and the hyporeflective area of the outer choroid. Because the hyperreflective areas correspond to the choroidal stroma and the hyporeflective areas to the choroidal vessels, these results are consistent with the earlier studies which reported that the increased choroidal thickness in eyes with CSC was associated with the hyperpermeability of the choriocapillaris and dilation of the larger choroidal vessels [2, 10, 39].

We reported earlier that the hyporeflective area but not the hyperreflective area in the outer choroid in the CSC eyes was significantly larger than that of the fellow eyes and normal eyes [33]. In addition, the hyporeflective area in the outer choroid in the fellow eyes was significantly larger than that in normal eyes. On the other hand, the hyperreflective area but not the hyporeflective area in the inner choroid of the CSC eyes was significantly larger than that of the fellow eyes, and the hyperreflective area in the inner choroid in the fellow eyes was not significantly different from that in normal eyes. These results suggest that the increase in the hyporeflective area of the outer choroid which corresponds to the dilation of the larger choroidal vessels may be a basic characteristic of the CSC disease process. In addition, the increase in the hyperreflective area of the inner choroid which corresponds to the swelling of the stroma may be the subsequent pathological change in eyes with CSC. The results of this study regarding chronic CSC at baseline confirmed the findings of our previous reports on acute CSC [33].

After the hPDT, the CCT and total choroidal area of the whole choroid were significantly decreased, and these changes were associated with a decrease in the hyperreflective area of the inner choroid and the hyporeflective area of the outer choroid. Chan et al reported that choroidal permeability and dilation of the larger choroidal vessels were decreased in the IA images after PDT in eyes with CSC [16]. Histopathological study of eyes with CSC in animals showed damage of the endothelial cells of the choriocapillaris and extensive leakage of fibrin into the stroma of the inner choroid [40]. One of the characteristic histopathological changes following PDT in human eyes is the occlusion of the choriocapillaris [41]. Our results are consistent with the IA and histopathological findings after PDT. It is believed that reducing the choroidal hyperpermeability is the main mechanism for the effectiveness of PDT on the serous retinal detachment in eyes with CSC [16]. The decrease in the hyperreflective area of the inner choroid may represent the decrease in the stromal swelling associated with the decrease in the hyperpermeability of choriocapillaris after hPDT. Layer-by-layer analysis was needed to determine the consistency between the morphological changes in the EDI-OCT images and the changes in the IA images and histopathological findings because the hyperreflective area of the whole choroid was not significantly changed after hPDT.

Although the mechanism causing the reduction of the hyporeflective area in the outer choroid was not determined, one possible explanation could be that the decrease in the hydrostatic pressure that results from the decrease in the swelling of the stroma reduced the peripheral vascular resistance, which then relieved the congestion of the choroidal vessels. Another explanation may be that the decrease in the hyporeflective area of the outer choroid was accompanied by a decrease in the fluid-filled spaces of the outer choroid. Spaide and Ryan reported that there were hyporeflective areas of fluid loculation in the outer choroid of CSC eyes [34]. Further studies using multimodal imaging that can evaluate the choroidal circulation with improved OCT technologies are needed.

We recently proposed a CSC index to represent the activity of the CSC. The index increases along with an increase in the size of the hyperreflective area of the inner choroid and the hyporeflective area of the outer choroid. In fact, the index was largest in eyes with active CSC with a SRD, intermediate in the fellow eyes of patients with unilateral active CSC, and lowest in normal eyes [33]. In this study, the index was significantly larger in the preoperative treated eyes than in the fellow eyes at the baseline, and decreased significantly at 1 month after the hPDT and continued to decrease until 12 months. However, the index at 12 months was significantly higher than that in normal controls. These results suggest that the structural abnormalities of choroid persisted even after the hPDT resolved the SRD at 12 months. The ratio of hyporeflective area to the total choroidal area of whole choroid was also decreased after the hPDT, but it may be less sensitive to the activity of the disease than the CSC index. At the baseline, the significance of the differences between the CSC eyes and the fellow eyes were larger in the CSC index (*P* <0.001) than in the ratio (*P* = 0.030). In addition, the index of the CSC eyes at 12 months was significantly larger than the normal control eyes (*P* = 0.002), but the ratio of the CSC eyes at 12 months was not significantly different from that of the normal control (*P* = 0.116).

At the baseline, the central choroidal thickness, total choroidal area, and hyporeflective area of whole choroid were significantly larger in the eyes with intense hyperfluorescence in IA images than in the eyes with intermediate hyperfluorescence. This may be because the activity of the disease in eyes with intense hyperfluorescence may be higher than that in the eyes with intermediate hyperfluorescence.

There are some limitations in this study. First, a retrospective study can have sampling biases. Second, the sample size was small. Third, the follow-up period of 12 months may have been too short, and only 22 eyes (75.9%) completed the 12 months of follow-up. Lastly, manual segmentation of the inner and outer choroid is not completely objective. However, to the best of our knowledge, this is the first study that evaluated the changes in the choroidal structures in eyes with CSC before and after the hPDT using layer-by-layer analyses.

In conclusion, the subfoveal choroidal area was significantly decreased after hPDT, which was most likely due to a decrease in the exudative changes in the inner choroid and a reduction in the dilation of larger vessels in the outer choroid.

## Supporting Information

S1 Table Raw data of choroidal parameters at each visit. (XLSX)

S2 Table Demographic data and the CSC index of age-, sex-, and refractive error-matched normal subjects. (DOCX)
