# Supplementary material for: Changes in Choroidal Structures in Eyes with Chronic Central Serous Chorioretinopathy after Half-Dose Photodynamic Therapy
Source: PLoS One. 2016 Sep 16;11(9):e0163104. doi: 10.1371/journal.pone.0163104 (PMC5026334; doi:10.1371/journal.pone.0163104)
Supplement: S2 Table — (DOCX) [file pone.0163104.s002.docx]

**S2 Table. Demographic data and the CSC index of normal subjects.**

|  | Mean ± SD | Range |
| --- | --- | --- |
| Sex | 22 men / 7 women |  |
| Age (yrs) | 59.4 ± 11.78 | 41 - 77 |
| Refractive Error (diopter) | -0.78 ± 2.16 | -6.0 - +2.5 |
| Hyporeflective / Total choroidal Area of whole choroid (%) | 65.2 ± 3.28 | 58.3 – 69.9 |
| CSC index | 0.99 ± 0.37 | 0.39 - 1.86 |

CSC, central serous chorioretinopathy; SD, standard deviation
